# Retraction: Potent Anti-Proliferative, Pro-Apoptotic Activity of the Maytenus Royleanus Extract against Prostate Cancer Cells: Evidence in *In-Vitro* and *In-Vivo* Models

**DOI:** 10.1371/journal.pone.0297383

**Published:** 2024-01-11

**Authors:** The PLOS ONE Editors

Following the publication of this article [1], concerns were raised regarding results presented in Figs 2, 3, 4, 5, 6, and 7. Specifically:

There are multiple instances in which western blot panels in [1] representing different experiments appear highly similar:

- Fig 3B 22Rν1 Cdk-2 and Fig 3C Cyclin D1
- Fig 3C 22Rν1 Cyclin E and Fig 3D 22Rn1 Bcl-XL
- Fig 3C C4-2 GAPDH and Fig 5A 22Rν1 GAPDH when rotated 180°
- Fig 4B C4-2 Caspase 3 (cleaved) and Fig 4V 22Rn1 Caspase 3 (cleaved)
- Fig 6B GAPDH and Fig 6D GAPDH when rotated 180°

Several images reported in [1] appear to have been reused in an article [2] published later by a different group:

- The following colony formation assay panels appear similar:
  ○ Fig 2B 22R1 Control of this article [1] and Fig 1C HCT116 Control of [2]
  ○ Fig 2B 22R1 40µg/ml of this article [1] and Fig 1C HCT116 40µg/ml of [2]
  ○ Fig 2B 22R1 60µg/ml of this article [1] and Fig 1C HCT116 60µg/ml of [2]
  ○ Fig 2B C4-2 Control of this article [1] and Fig 1D HT29 Control of [2]
  ○ Fig 2B C4-2 40µg/ml of this article [1] and Fig 1D HT29 40µg/ml of [2]
  ○ Fig 2B C4-2 60µg/ml of this article [1] and Fig 1D HT29 60µg/ml of [2]
- The following immunohistochemistry microscopy panels appear similar:
  ○ Fig 5B C4-2 Control of this article [1] and Fig 7C of [2]
  ○ Fig 5B C4-2 MEM of this article [1] and Fig 7D of [2]
  ○ Fig 5B 22Rν1 Control of this article [1] and Fig 7A of [2]
  ○ Fig 5B 22Rν1 MEM of this article [1] and Fig 7B of [2]
- The following western blot panels appear similar:
  ○ Fig 3C C4-2 Cyclin B1 of this article [1] and Fig 6A HT29 β-catenin of [2]
  ○ Fig 4C C4-2 Bid of this article [1] and Fig 4A HCT116 Bid of [2]
  ○ Fig 4C 22Rn1 Bid of this article [1] and Fig 4A HT29 Bid of [2]
  ○ Fig 4D C4-2 Bcl-2 of this article [1] and Fig 5 HCT116 Bcl-2 of [2]
  ○ Fig 4D 22Rn1 Bcl-XL of this article [1] and Fig 5 HT29 Bcl-xL of [2]

PLOS was unable to clarify the origins of the images that appear to be reused between [1] and [2].

Regarding the similarities observed between western blot panels in [1], the first author disagreed with the editorial assessment and stated that there are differences between the blots. The *PLOS ONE* Editors remain concerned that the indicated blots appear more similar than would be expected from independent results. The first author provided some underlying data, but they did not resolve the concerns.

In light of the similarities between multiple western blot panels presented in this article [1] that question the reliability and validity of these data, the *PLOS ONE* Editors retract this article.

MS did not agree with the retraction. DNS, RKL, MRK, and HK either did not respond directly or could not be reached.
